# Supplementary material for: Categorical representation from sound and sight in the ventral occipito-temporal cortex of sighted and blind
Source: eLife. 2020 Feb 28;9:e50732. doi: 10.7554/eLife.50732 (PMC7108866; doi:10.7554/eLife.50732)
Supplement: Supplementary file 1. [file elife-50732-supp1.docx]

**SI Table 1. Categories and stimuli.**

| CATEGORIES | STIMULI |
| --- | --- |
| BIRDS | Canary  Owl  Seagull |
| MAMMALS | Dog  Donkey  Horse |
| HUMAN  VOCALIZATIONS* | Woman  Man  Man |
| HUMAN  NON VOCALIZATIONS | Women laughing  Man crying  Women yawning |
| TOOLS | Hairdryer  Saw  Toothbrush |
| GRASPABLE  OBJECTS | Guitar  Keyboard  Telephone |
| BIG MECHANICAL  OBJECTS | Church-bell  Traffic  Train |
| ENVIRONMENTAL  SCENES | Storm  River  Wind |
| **Neutral faces in the visual experiment* | |
